# Early Probe and Drug Discovery in Academia: A Minireview

**DOI:** 10.3390/ht7010004

**Published:** 2018-02-09

**Authors:** Anuradha Roy

**Affiliations:** HTS Laboratory, University of Kansas, Lawrence, KS 66045, USA; anuroy@ku.edu; Tel.: +1-785-864-1709

**Keywords:** high-throughput screening, assay development, small molecules, drug and probe discovery

## Abstract

Drug discovery encompasses processes ranging from target selection and validation to the selection of a development candidate. While comprehensive drug discovery work flows are implemented predominantly in the big pharma domain, early discovery focus in academia serves to identify probe molecules that can serve as tools to study targets or pathways. Despite differences in the ultimate goals of the private and academic sectors, the same basic principles define the best practices in early discovery research. A successful early discovery program is built on strong target definition and validation using a diverse set of biochemical and cell-based assays with functional relevance to the biological system being studied. The chemicals identified as hits undergo extensive scaffold optimization and are characterized for their target specificity and off-target effects in in vitro and in animal models. While the active compounds from screening campaigns pass through highly stringent chemical and Absorption, Distribution, Metabolism, and Excretion (ADME) filters for lead identification, the probe discovery involves limited medicinal chemistry optimization. The goal of probe discovery is identification of a compound with sub-µM activity and reasonable selectivity in the context of the target being studied. The compounds identified from probe discovery can also serve as starting scaffolds for lead optimization studies.

## 1. Introduction

High-throughput screening (HTS) is an essential enabling technology for translational research that can have endpoints of drug discovery or probe discovery [1]. The end point of drug discovery is a complex process that leads to identification of a drug candidate that has potential for becoming a marketed drug. Drug discovery is both a cost- and time-intensive process that requires integration of expertise from various specialized teams and can take up to 15 years to bring a candidate molecule to the market. Despite multi-million dollar investments in research and development (R&D) and in implementing regulations, drug discovery and development is a very risky process for big pharma. Risks arise from clinical trial failures due to lack of drug–target engagement, lack of correlation between target and disease, and inadequate endpoint and patient selection. Other risks include patent expirations and competition from generics, drug discontinuation due to long-term safety issues, and poor efficacies across much larger genetically diverse populations. The high costs and risks and long timelines of real world drug discovery are not compatible with the much shorter project milestones and small research budgets of the academic world [2]. Notable exceptions to this generalization include academic labs that pursue comprehensive early and pre-clinical drug discovery research programs. The second endpoint of translational research, probe discovery, is a more viable alternative in academic settings [2,3]. Probe discovery can be viewed as an intermediate short-term process of compound identification, where the candidate molecule is used as a tool to dissect a biological process or pathway of interest. The process of probe discovery is well suited to operating within limited budgets, capturing short-term milestones defined by probe discovery publications and new grant submissions. Probe discovery also accommodates the vast array of targets and biological systems that academics pursue regardless of commercial return on investment value.

## 2. Target Identification and Validation

Regardless of whether the goal of translational research is drug or probe discovery, both endpoints require the identification of a target or a pathway via basic academic or clinical research. Studies on the molecular mechanism of disease unravel targets that are relevant to disease development and progression [4]. The targets range from proteins, mutations, or polymorphisms in the coding or non-coding regions of the genome or transcriptional or post-translational regulatory processes. At one end of the target spectrum, defining the role of target is relatively clearer in simple mono-factorial diseases, which are characterized by one causative allele, and modulating that single gene or factor theoretically increases the probability of targeting the disease effectively. At the other end of the target spectrum, defining a target in complex multifactorial diseases is extremely challenging. Complex diseases, including cancer, neurodegenerative disorders, and Type 2 diabetes, are all heterogeneous and have variable phenotypes from risk factors that are a function of genetics, age, gender, and diet or lifestyle choices. The challenges in identifying a unique target underlying complex diseases arise from cellular cross-talks between signaling pathways and interaction networks that result in functional redundancies and other compensatory mechanisms. As the industry strives to constantly improve its capabilities and predictive powers in drug discovery to produce safer and more efficacious drugs, it has become very clear that target information appears to fall short in many cases in late-stage drug discovery. The importance of acquiring more comprehensive information on targets of interest cannot be understated.

A target is deemed druggable when it is amenable to modulation either through genetic and/or chemical experimentation. Modulation of the target should elicit a measurable and quantifiable response, which in turn establishes a strong, unequivocal relationship between the target and disease development or progression. The field of target identification mechanisms has been reviewed extensively [5]. In short, the functional significance of a target in disease or pathogenesis can be measured through the genetic manipulation of cells or animal model organisms using clustered regularly interspaced short palindromic repeats (CRISPR-Cas9), transcription activator-like effector nucleases (TALENs) and zinc-finger nucleases (ZFNs), RNA interference or microRNAs. Chemical validation of the target can be performed using known compounds or available antibodies. The probability of identifying a molecule that has high potential of engaging a target effectively requires strong target validation data and helps in overall risk assessment [6]. The success of designing relevant assay systems requires characterizing the target in both normal and pathogenic states: sequence and structure, functional or structural redundancy, characterization of spliced isoforms, posttranslational modifications, subcellular distribution, and mRNA and protein expression levels across tissues, their half-lives, and regulation. Target deconvolution can also be attempted using mass spectrometry following thermal protein stabilization (CETSA) [7] or affinity enrichment techniques in the presence or absence of a drug, followed by genetic validation.

Target identification in recent years can also be supported from extraction and integration of relevant information available from different datasets [8]. Extensive research and clinical observations over the years have made available large volume “omics” datasets, which may prove critical in target and drug discovery. Correlative integrations across omics databases are presumed to facilitate the building of more comprehensive models of targets in disease. The high-throughput genomics, transcriptomics, epigenomics, proteomics, and metabolomics can help establish strong gene/protein variant associations and can aid in biomarker discovery. Ultimately, models emerging from strong correlative datasets may help identify correct patient populations and define relevant clinical endpoints for the diagnostics, prevention, or treatment of diseases.

## 3. Assay Development

Probe or drug discovery requires development of a primary screening assay that faithfully interrogates the target or pathway being studied. In addition to the primary assay, the screening workflows also require secondary assays for hit characterization [9]. The assays can be developed using biochemical or cell-based platforms. Biochemical assays are targeted assays that have well-defined reaction components that are incubated with purified compounds to find inhibitors or activators. Biochemical assays include enzymatic assays (direct or coupled) run as end-point or kinetic assays, protein–protein or protein–DNA or protein–RNA interactions, or direct compound binding assays. Cell-based assays range from being black box assays based on generic phenotypic read outs like cytotoxicity or can be relatively more targeted, as in biomarker-based screens. Cell-based reporter gene assays are built up on target sequences fused to reporters such as luciferase, β-galactosidase, and fluorescent proteins. Phenotypic assays measure phenotypic or biochemical changes in cell lines, primary cells, or zebrafish, *Caenorhabditis elegans* model organisms. Unlike the biochemical assay readout, defining the mechanism of action of a compound identified through whole cell assays is more complex. Cell-based assays are considered more mechanism-based if the screening readout is a direct measure of target engagement, as in in-cell Western assays, split-protein complementation (biomolecular luciferase or two hybrid assay systems), or using labeled substrates or indicators of cellular enzymatic or translocation screens. The cell-based assays developed for high content imaging are highly informative and can simultaneously provide information on cell morphology, integrity of nucleus, cytoplasm or membranes in addition to other targeted processes being measured, e.g., changes in intracellular localization or accumulation of lipid vesicles in algae. The relevance of the type of cells used for developing a primary screen or secondary assays is critical for identification of a specific probe/drug. The screens involving stem cells, patient-derived cells [10], and three-dimensional spheroids formed from single cell lines or formed by mixing various organ-specific or tissue-specific cell types has greatly expanded the capabilities of finding more relevant and translatable hits in cancer and hepatocyte injury mechanisms. Many biochemical assays as well as cell-based reporter systems usually contain smaller truncated functional domains of regulatory elements, proteins/enzymes for convenience of purification or for improvement in signal windows. Compounds active against such truncated targets may also need to be tested against full-length proteins or under more physiologically relevant conditions.

In addition to the primary assay, probe and drug discovery work flows require setting up additional assays that will be very useful downstream to characterize the compounds for reconfirmation of activity as well as for defining selectivity and specificity [11]. The orthogonal assays confirm the activity of compounds identified from the primary screen, while secondary assays that target other known related members of a protein family or other relevant proteins will help define compound selectivity. The orthogonal assays serve to confirm the activity of primary screening hits against the target and eliminate compounds that interfere non-specifically with the primary assay components or detection methodologies. For instance, while the luminescence-based Alpha screen assay can be used to screen for compounds that inhibit protein–protein interactions, an assay based on Homogeneous Time Resolved Fluorescence (HTRF) serves as an orthogonal assay to characterize the interactions using fluorescence-based technology. Whenever possible, it is good practice to include both biochemical and cell-based assays for the target or signaling pathway being investigated. The hits from biochemical assays should be tested for their permeability across cell membranes and their ability to modulate the target inside the cells. Likewise, the compounds identified from cell-based assays should be tested for their ability to bind to the target of interest using biochemical or biophysical assay read outs. While a compound that shows high potency in a biochemical screen but not in cell-based assay and vice versa can still be pursued as there is always a chance that medicinal chemistry optimization can help modify the physicochemical properties of the hit and make it more useful. In general, compound screening work flows designed to include biochemical, biophysical, and cell-based assays provide better insight into the quality of hits and provide support for data-driven hit selection for downstream processes.

## 4. Assay Optimization

**Figure 1:**
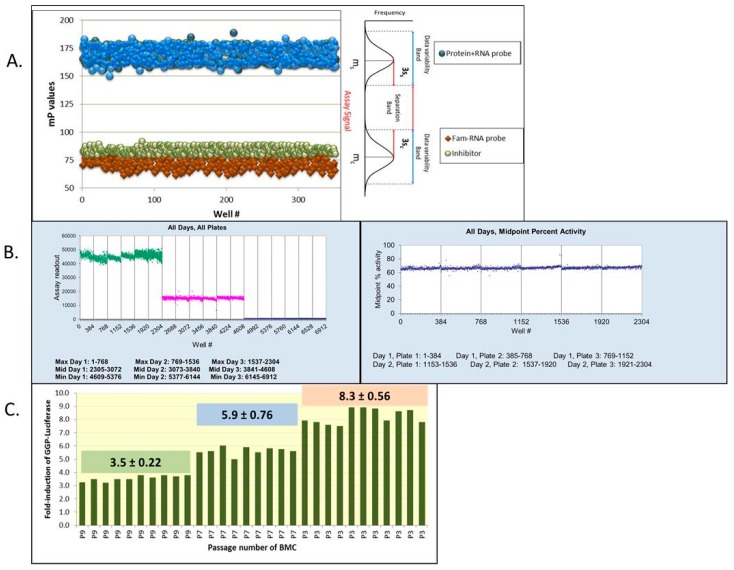
Representative assay optimization parameters. (**A**) Scatterplot of signal separation between the high (RNA + protein) and low (RNA alone) polarization values, giving an acceptable band separation and average Z’ values of 0.8. (**B**) Plots showing signal consistency in 3-day, 3 plate assay and mid-point percent activity from inter- and intraday assays. The well numbers are shown on x-axis of the plots. (**C**) Effect of passage number of chemical inducer of dimerization (CID)-dependent bone marrow cells (BMC) on γ-globin promoter driven (GGP)-luciferase induction in the cell-based reporter assay system.

For any system under consideration, it is the biology and practicality that dictates the type of assay selected for high throughput screening. The assays developed for high throughput screening are preferably homogeneous, involving up to two steps for their execution. Heterogeneous formats requiring multiple additions and removal of reagents add to variability in signals when large number of assay plates are processed. The assays should be scalable and able to be miniaturized from 96-well formats to 384–1536 high-density well microplate formats with no significant loss of response. The assays for screening should be highly reproducible and robust. The same signal windows between low and high counts from plate to plate and from day to day must be exhibited in positive and negative controls (Figure 1A,B). A comprehensive guide for assay development is available from NCBI [12].

The availability of large amounts of purified proteins, labeled peptides or nucleic acids, affinity of interactions, the stability of reagents over time are some of the factors impacting biochemical assay set ups. Cell-based assays require an understanding of the impact of passage number on assay readout (Figure 1C) and on cell physiology as well as the impact of expressing reporters or overexpressing proteins on cell metabolism, growth kinetics, and gene expression. The cell lines being used should be authenticated via short tandem repeat (STR) PCR analysis and be routinely screened for mycoplasma. The assays are developed using any of the available platforms including fluorescence (fluorescence intensity, polarization, Förster resonance energy transfer (FRET), and Time-resolved fluorescence energy transfer (TR-FRET)), absorbance, luminescence, Bioluminescence resonance energy transfer (BRET), Alphascreen technology, and label-free assay systems. For any new assay, a plate uniformity and signal variability test is performed using conditions that generate a Min, Mid, or Max signal values for any of the platform technologies. The signal reproducibility and signal separation between the conditions generating high, medium, or low signals are tested by independently setting up three plates per day over a period of three days. The “Max” signal measures the high read outs from reactions or cells containing agonists or DMSO vehicle. The “Min” signal is the basal signal read from a biochemical reaction that lacks an enzyme, or has a low concentration of agonist, or reactions with high concentration of an inhibitor, all of which can reduce- the signal-output to > 80% of the untreated response. The data is used to evaluate patterns of drifts and edge effects across rows and columns of each plate. The inter- and intra-day tests measure shifts, if any, in the Mid signal, normalized to Max and Min signals, signal windows, and a coefficient of variability (CV) (Figure 1B). An acceptable assay shows SW > 2, CV_Max_ and CV_min_ < 20%, and a normalized midpoint signal shift of <2. The distribution of Max and Min signals or of high and low controls are also used to calculate a statistical screening window factor called the Z factor. The Z’ factor is based on means and standard deviations of controls (Z’ = 1 − (3SD_high control_ + 3SD_low control_)/|Mean_high control_ − Mean_low control_|. During screening, the quality assessment on screen samples is used to calculate the Z factor, which is based on means and SD of sample and negative controls (Z = 1 − (3SD_smaple_ + 3SD_low control_)/|Mean_sample_ − Mean_low control_|. While the majority of biochemical and cell-based screens yield acceptable Z’ scores, the screening positives in RNA interference-based screens are more noisy and can generate moderate to weak effects, yielding a lower signal to noise windows and failing Z’ scores. Other statistical criterion has been published (Strictly Standardized Mean Difference (SSMD), Receiver Operating Characteristic curve (ROC) curves, Mean + standard deviation threshold (k), etc.) for defining quality standards of RNAi screens [13,14]. More detailed discussion on practical aspects of establishing statistical criterion measurements that certify the readiness of an assay for screening large compound collections can be obtained from the NCBI [12]. The siRNA-based screening optimization is discussed in detail in references [13,14]. The assays are transferred from bench top to automated robotic systems for compound and liquid dispensing operations. The automation systems range from pin-tool tips to acoustic transfer capabilities, the availability of which depends largely upon the academic facility budgets and their optimal workflows.

## 5. Compound Screening Process

Once the assay meets the established statistical acceptance criterion for screening (e.g., signal windows, coefficient of variability, reproducibility, Z’ factor, uniformity, etc.), the assay is first used for screening a small training set of compounds (~2000–10,000), to verify that the assay is performing acceptably. The test compound collections can vary with the screening facility or can be dictated by the target class. The validation or test library can comprise a collection of small molecules representative of scaffolds present in much larger compound sets, may include a collection of known bioactives that include compounds with at least one known molecular target, and may include kinase or phosphatase inhibitors or inhibitors of a cell cycle, proteasomes, etc. The data from the validation screening is evaluated for various parameters like the Z’ scores, signal uniformity, hit rates, frequency of false positives, and assay interference compounds.

**Figure 2:**
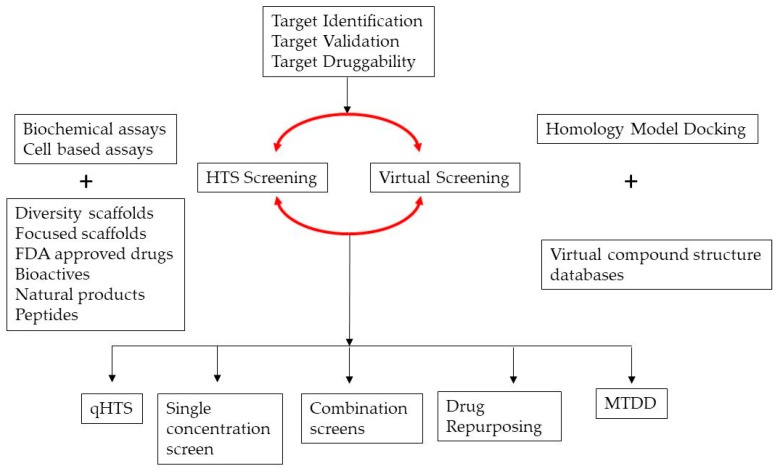
Steps in early drug/probe discovery. Translational programs require stringent target identification and validation information. Druggable targets are screened either virtually using virtual compound structure databases or via biochemical or cell-based assay screening of available chemical or peptide libraries. High throughput Screening (HTS) can be performed with single purified compounds at one or more concentrations (qHTS). Combination screens are performed to identify synergistic combinations of bioactives. Drug repurposing screens identify new targets for FDA-approved drugs, while multi-target drug discovery (MTDD) approaches identify compounds with activity against two unrelated targets.

Once the assay passes all validation screening parameters, larger compound collections are screened at one or more concentrations. The primary screening can be performed at a single concentration or can be performed at 6–8 concentrations in quantitative HTS format (qHTS) (Figure 2). The qHTS, though more cost- and time-intensive, helps in generating dose–response curves for each of the compounds tested. The qHTS format also minimizes the selection of false positive hits. Combination screening is yet another mode of screening in which the synergistic combination of compounds is identified by combining either single or multiple concentrations of compound or drug of interest with other drugs or bioactives or diversity scaffolds from focused libraries. Combination screening can also be performed for drug repurposing projects where a clinical standard of care is used to screen other US Food and Drug Administration (FDA)-approved drug collections to identify more potent synergistic combinations of drugs for new indications or for improving efficacy of existing standard of care. New combinations may help identify two or more drugs that target multifactorial disorders and improve the quality of care. In addition to the wet bench HTS campaigns, virtual screening can also be performed to select primary hits. The confidence of hit identification via virtual screens increases if the screening model is supported by X-ray crystal structures of binding domains or co-crystal models with ligands. The challenges of defining a theoretical low energy model in the absence of crystal structure information makes true positive identification from virtual screening more challenging. In contrast to HTS, which is dependent on actual available compound libraries, virtual screens can access much larger compound collections from databases.

Minimal chemical scaffolds can show binding promiscuity and can modulate several targets that share functional domains and binding sites across target families. The multiple target modulation by a drug or polypharmacology is exploited in screens for drug repositioning that help in the identification of new indications for marketed drugs. Identification of new activity for a marketed drug via experimental and in silico-based approaches fits well within the scope of academic discovery projects, as the safety, toxicity profiles, formulations, and pharmacology of marketed drugs are already established. A combination of two or more FDA-approved drugs against a target may improve outcomes especially if the combinations target the crosstalk between pathways that are activated or repressed in disease settings. Another form of multi-target drug discovery (MTDD) screening involves identification of single compounds with activity against two or more targets that reside in the same tissue or cell compartment. In such screens, the hit compounds identified from the first screen against a target are used against the second target of interest to identify scaffolds with activity against both targets. Both experimental as well as virtual in silico approaches can be used to design screens to identify compounds that are active against multiple targets of interest. Several rationally based designs, computationally based docking, and virtual screening approaches are available for identifying drugs with multiple functions. The prediction of interactions between a chemical compound and other potential biological targets require a mining of “omics” datasets, molecular docking using X-ray crystal structures or models, ligand-based quantitative structure–activity relationship (QSAR) similarity prediction of two- or three-dimensional fingerprints of small molecules, and binding pocket subcavities that have been shown to accommodate known drugs across proteins that lack sequence similarity [15]. Polypharmacology-based screens can help in the selection of molecules that have higher efficacy and lower toxicity.

## 6. Screening Libraries

The assay interrogating a target is used to screen collections of chemicals or nucleic acids or peptides. The chemical collections or libraries include fragments ((<300 Da), small organic molecules (300–500 Da) and macrocycles or larger structures of high molecular sizes. Libraries of small organic molecules are used most extensively in small molecule high throughput screening campaigns. The chemical libraries are either available commercially through vendors or are chemical collections synthesized by academic scientists. The libraries may be available as diversity scaffolds that generally follow Lipinski’s Rule of Five (Ro5) concept for bioavailability. The Ro5 criterion leads to the selection of molecules that are <500 Da, have <5 H bond donors (N–H & O–H bonds), have <10 H bond acceptors (N + O), and have a octanol–water partition coefficient logP <5 [16]. The compound collections are routinely screened with filters to eliminate chemical liabilities or synthetically challenging scaffolds. The filters can remove scaffolds with known reactivities, detergents, denaturing agents, oxidizing/reducing agents, etc. or pan assay interference compounds (PAINS), which are associated with promiscuous activity or possess assay interference attributes [17]. Several filters such as Rapid elimination of Swill (REOS), Lilly Rules [18] and PAINS filters are currently available, and, when used in parallel, the filters collectively can flag from 5 to 60% of the vendor library compounds (personal communication). Scaffold collections are also selected via in silico design based on known bioactives to form focused libraries against popular targets like ion channels, kinases, Hsp90, proteases, anti-fungal, or anti-infective properties or selected in silico for having properties to cross the blood–brain barrier. Several reports indicate that the vast majority of approved drugs belong to small classes of organic molecules and that half of all bioactivity is associated with less than 5% of the known chemical scaffolds. A much larger chemical space is represented by fragment collections, which follow the rule of three (Ro3) criterion [19,20]. Fragments are selected based on a molecular weight <300, having ≤3 H bond acceptors, having ≤3 H bond donors, and having a clogP <3. Fragment-based screening is generally performed using NMR-based or Biacore- or X-ray crystallography driven screening platforms. FDA-approved drugs as well as known bioactives comprise yet another type of screening sets that can be utilized for drug repurposing screens [21]. The FDA-approved drugs provide an opportunity to fast track the discovery process as the pre-clinical and clinical safety data is already available [22]. The partial coverage of chemical space has also supported expanding screening to purified natural products and into exploring compounds from rich biodiversity in terrestrial and marine organisms. The problems of purification and scale-up production of active scaffolds from natural extracts limit access to large natural product collections that are available for screening.

In addition to the chemical libraries, genomic screening uses collections of siRNAs [23], microRNAs [24], or CRISPR libraries [25]. Genomic screens provide valuable target and mechanistic information but can often result in complex datasets. Phenotypic assays are used to screen biologics, which have proven to be highly effective therapeutic agents, and the path to their discovery is distinct from that followed for small molecule development. The biologics include recombinant protein-based therapies such as monoclonal antibodies, growth factors, hormones, vaccines, and anticoagulants that are produced in bacteria, yeast, and mammalian cells [26]. Peptide-based drug discovery [27] is yet another major area of screening but has its own challenges in terms of their stability and availability. In addition to the above agents, stem cells by themselves hold great promise as novel therapeutic agents but require precisely defining human cell lineages, identifying markers, niche-dependent potency and process-controlling proliferation, differentiation, and functional specialization. Gene expression-based RT-PCR (reverse transcription-polymerase chain reaction) screens are also used to identify compounds that can help maintain the self-renewal potential of stem cells. Such screens help in identifying hits that directly or in combination with genetic factors, can support generation of induced pluripotent- and lineage-specific stem cells from somatic cell types.

## 7. Actives: Hits to Leads

“Actives” are compounds that show activity in the primary assay. The single concentration primary screens are followed by activity reconfirmation in up to a ten-concentration dose–response. Since the primary hit rates can range from 0.1% (for some biochemical screens) to as high as 4% for some cell-based screens, it is useful to include a selectivity and/or cytotoxicity assay at this stage of hit identification. The dose–response curves are analyzed for their quality (Hill slopes, potency, and efficacy). At this stage, the reconfirmed hits are also evaluated for their promiscuous activities across other known screening datasets. These include internal HTS databases as well as large data collections in the PubChem database [28]. Compounds with activities against other targets or known assay interference characteristics are culled or deprioritized and a relatively selective set of hits from the screen are subjected to cheminformatics analysis [29,30,31]. Cheminformatics analysis utilizes multiple approaches based on Tanimoto coefficients, daylight substructural fingerprints, or modified Jarvis–Patrick non-hierarchical cluster analysis [32] to group the hits into several structure–activity clusters. The cheminformatics datasets are analyzed by medicinal chemists for structural features for easy chemical manipulation, and a few representative compounds from each cluster are re-purchased as fresh powders from vendor catalogs or synthesized in-house. Repurchasing new powders ensures that the observed activity corresponds to a unique intact scaffold and is not due to some impurity or degradation or transformed scaffold in DMSO stocks, which, in HTS labs, undergo several freeze–thaw cycles over time. The purity of repurchased/synthesized compounds is established by liquid chromatography–mass spectrometry (LC-MS) analysis. The activity from the fresh powders that are >95% pure is again tested in the primary screening assay as well as other follow-up secondary assays (selectivity screens, cytotoxicity studies, activity in a physiologically relevant assay, distinct from the primary assay). A proof of concept data for early discovery is generated, and, at this stage, analoging by catalog or by synthesis is initiated, or grants are submitted for acquiring funding for further development of the scaffolds.

To ensure that the hits are not false positives that interfere with assay detection methodologies, the hits are validated using orthogonal assays based on detection technology distinct from the primary screening assay. At this stage, a wide range of low to medium throughput assays are employed to further characterize the hits. Some of these assays include RT-PCR, Western blot analysis, cytotoxicity assays to establish mechanism driven cell lethality, target selectivity assays for screening across a panel of orthologous targets or for infectious diseases, and a panel of Gram-negative or Gram-positive bacteria. Mechanistic studies are greatly encouraged to define mode of action.

The molecular scaffolds from iterative medicinal chemistry optimization result in the identification of “probe” molecules, which show high potency (<100 nM for biochemical screen, <1–10 uM for cell-based assays), high efficacy (maximal effects in physiologically relevant systems), and good selectivity (ranging from >10 fold difference to >100 fold, depending upon the biological system) [33]. High quality probes are also expected to exhibit good aqueous solubility and membrane permeability properties [34].

**Figure 3:**
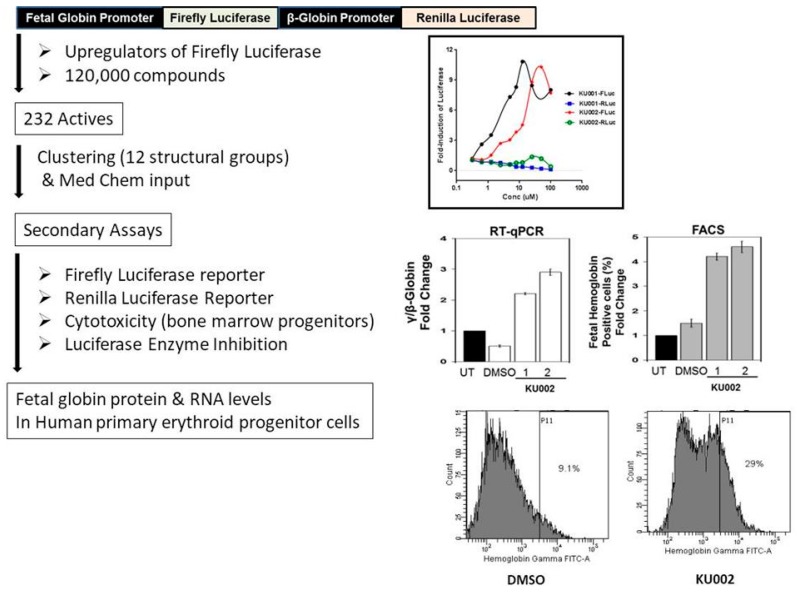
Screening for upregulators of fetal globin promoter targeting Sickle Cell disease. A dual luciferase construct was used to screen 120,000 compounds. Representative compounds from 12 structural clusters were tested in secondary screens to define compound activity. Two of the most potent actives preferentially upregulated firefly luciferase but not Renilla luciferase, were not cytotoxic to bone marrow cells (BMPs) and did not inhibit purified luciferase enzyme in biochemical assay. The compounds showed significant induction of γ-globin RNA and protein as evidenced from qRT-PCR and fluorescence-activated cell sorting (FACS) analysis in human primary erythroid cultures.

A simple screening tier is shown in Figure 3. The overall goal of the project was the identification of small molecules that reactivate expression of γ-globin (HbF, fetal hemoglobin), which has therapeutic significance in the treatment of sickle cell anemia [35]. The assay utilized immortalized multipotential cells derived from the bone marrow of transgenic mice, stably expressing a dual luciferase construct, with firefly luciferase under fetal globin promoter and Renilla luciferase under the control of the β-globin promoter. The screening parameters including cell passage numbers were optimized in 384-well format, and the cell lines were characterized for their ability to respond to at least 10 known inducers of fetal globin-like hydroxyurea, sodium butyrate, valproic acid, valeric acid, etc. The assay was used to screen 120,000 compounds from the University of Kansas HTS compound collection and 232 compounds were found to upregulate firefly luciferase after 24 h of incubation. The actives were clustered into 12 structural groups and fresh compounds were repurchased from various vendors. Three cell-based secondary assays were performed using the freshly available compounds for their ability to modulate firefly luciferase, Renilla luciferase, and general cytotoxicity in bone marrow progenitor cells. The activity of compounds was also tested against purified luciferase in an optimized biochemical assay. Profiling of the compounds revealed that at least 50% of the screen actives selectively upregulated firefly luciferase but did not upregulate β-globin promoter-driven Renilla luciferase activity. The compounds that selectively upregulated firefly luciferase activity also did not inhibit purified luciferase enzyme activity and were not toxic to bone marrow progenitor cells. The concentration–response curves show activity of two such compounds identified in a primary screen: K001 and K002 upregulated firefly luciferase up to 10-fold and did not upregulate Renilla luciferase activity. The most potent and selective compounds were tested for their effects on fetal globin RNA and protein levels using the primary human erythroid cultures generated in vitro from adult CD34+ stem cells. The erythroid stem cells were treated with compounds for 48 h and harvested for gene expression and fluorescence-activated cell sorting(FACS) analysis. The test compounds were found to induce a 2.8–2.9-fold increase in the ratios of fetal gamma to β-globin RNA level compared with 1.8-fold increases with known inducer sodium butyrate. FACs analysis showed a 2–5-fold increase in fetal globin positive cells. These results indicate that the actives identified in the high throughput screening assay using a reporter assay were also functional as HbF inducers under a physiologically relevant human primary erythroid cells.

## 8. Hit to Lead Optimization

Hit to lead optimization programs are executed to identify hit classes that are chemically tractable and serve as good starting points for developing quantitative structure activity relationships [32]. A more involved chemistry and absorption, distribution, metabolism and excretion (ADME) program is initiated for hit to lead optimization. Chemical optimization of generally from 2 to 10 scaffolds series for drug discovery are initiated to improve potency and efficacy and selectivity of compounds. The compounds acquired or synthesized through medicinal chemistry efforts are tested iteratively in a few selective assays. Selectivity screens are often expanded to include related target class panels or with unrelated targets. A simultaneous evaluation of in silico analysis of compound solubility, permeability, and toxicity profiles is performed. Molecular modeling and crystallization docking programs can be run in parallel for rational and informed compound analog design. Over the last few years, cellular thermal shift assays (CETSAs) have been a highly recommended choice for defining drug–protein interactions in cells in medium to high throughput formats [7,36]. In addition to their various applications, CETSAs are a powerful tool used not only to confirm the binding of the compound to target of interest but can also be used to define off-targets via mass-spectrometric analysis of compound-stabilized proteins in the cell. Using various approaches, the hit to lead optimization program results in the identification of new analogs with improved potency, high efficacy, reduced off-target activity, and >100-fold windows between non-specific cytotoxicity and target activity. Physico-chemical properties include testing analogs for permeability and absorption through Caco-2 cells, metabolic stability in rat/mouse and human microsomes, cytochrome P450 inhibition, human ether-a-go-go-related gene (hERG) channel inhibition, and plasma protein binding. Parallel improvement in physico-chemical and metabolism-related properties greatly improve the prediction of outcomes in subsequent pharmacokinetics and pharmacodynamics models. Lead identification results from developing highly optimized scaffold molecules with good drug-like properties, high efficacy and potency and with acceptable selectivity. The final lead scaffolds bear very low semblance to the original hit identified through screening chemical libraries. Toxicology studies in at least two animal models species as well as conformation to regulatory studies are performed at this stage. A thorough understanding of target biology enables a clearer understanding of the mechanism of action of the lead compounds. Target- and off-target binding studies help- in defining a mechanism of action of hits and also in the identification of other related or unrelated targets and signalling pathways. Information on tissue distribution of the hit molecule is critical for quantifying compound exposure at the site of action, whereas on-target occupancy helps to in defining drug efficacy and engagement time [37]. Assays and disease models that establish correlations between the functional effects of molecule–target interaction and a quantifiable and physiologically relevant phenotype in disease models are critical for validating role of targets and chemical molecules in disease.

## 9. Overall Impact of Academic Early Discovery Programs

Early discovery programs in academia have contributed significantly to both the fundamental processes and screening resources in early probe/drug discovery. Academic drug discovery programs started evolving at a time when the patents in big pharma settings were nearing expirations and the industry started engaging in more collaborative and open innovation discovery paradigms [38]. The new industry mind-set in combination with the enhanced translational focus of National Institutes of Health (NIH) led to the emergence of screening centers. The NIH-funded Molecular Libraries Probe Production Centers Network (MLPCN) helped set up comprehensive screening centers and specialized chemistry centers, which triggered academic screening programs. The pharma-dominated drug discovery endeavors were guided largely by Return on Investment (ROI)-driven business decisions that supported low risk projects affecting large patient populations, often leading to the discovery of “me-too” drugs. Academic drug discovery stepped in to fill the gaps in target discovery and in expanding the chemical space. Academic early discovery programs have helped identify targets for unmet medical needs, in almost all areas of human diseases, including cancer and many metabolic, neurological, and infectious diseases. The small molecule screening decks expanded due to novel compounds and novel synthesis protocols emerging from academic synthetic chemistry research [39,40,41], or from the NIH-funded Chemical Methodologies and Library Development (CMLD) programs [42,43,44]. Several new methodologies and approaches have been reported in literature for hit evaluation [6,18,29,45,46,47,48], tapping dark matter as starting leads [49], and shifting focus to covalent binders [50]. The NIH-MLPCSN programs have provided excellent guidelines to the academic community for adapting their target ideas into successful HTS assays. The screening grants based on the principal investigator’s biology have helped expand the scope of academic research, have supported screening and hit optimization projects of academic HTS and medicinal chemistry laboratories, and have supported the research infrastructure costs of universities and departments.

## 10. Summary

Chemical probes generated through academic programs can provide valuable information on target biology and translatability. The NIH Molecular Libraries Program contributed significantly towards probe discovery and developed 375 probes against a large number of targets [42]. The academic drug discovery consortium [51] (ADCC; [52]) lists 149 drug discovery centers across the world. While some of the screening centers focus on specific diseases or capabilities, a vast majority of the screening facilities work on a wide range of targets and diseases [53]. The screening centers differ in the size of screening resources (compound collections, automation equipment, platform detection technologies and capabilities) as well as in the extent of a university’s drug discovery infrastructure support. The extent to which an academic discovery project is taken along the course of mid- to late-stage discovery depends largely on resources and an interest in establishing multidisciplinary collaborations as well as on obtaining funds for medicinal chemistry optimization, ADME, pharmacokinetics and pharmacodynamics, formulations and toxicology are bottlenecks for majority of the academic programs. The most productive collaborations arise when the high quality probes developed in academic early discovery programs move further into the late-stage discovery workflows in pharma settings. Around 24% of FDA-approved drugs between 1998 and 2007 were reported to have emerged from transfer of scientifically innovative university patents to biotechnology and pharmaceutical companies [54]. Academic probe discovery programs have not only expanded the scope of academic basic research projects but have also brought in innovative approaches into traditional drug discovery processes.
